# Longitudinal Change in Peripheral Anterior Chamber Depth of Eyes with Angle Closure after Laser Iridotomy

**DOI:** 10.1155/2018/9106247

**Published:** 2018-12-24

**Authors:** Toshie Furuya, Kenji Kashiwagi

**Affiliations:** Department of Ophthalmology, University of Yamanashi, Chuo, Yamanashi, Japan

## Abstract

**Purpose:**

To investigate longitudinal changes in peripheral anterior chamber depth (pACD) of eyes with angle closure after laser iridotomy (LI) and factors related to prognosis.

**Design:**

Retrospective cohort study.

**Methods:**

Eyes with primary angle closure (PAC), acute PAC, or chronic angle closure glaucoma (CACG) that underwent LI (LI group) and eyes that underwent phacoemulsification and intraocular lens insertion (PEA + IOL group) were employed. Longitudinal changes in pACD were evaluated with a scanning peripheral anterior chamber depth analyzer (SPAC) in addition to routine ophthalmic examination.

**Results:**

Forty-eight eyes of LI groups (69.8 ± 8.5 years) and 21 eyes of PEA + IOL group (65.6 ± 12.7 years) were enrolled. Mean follow-up times of LI group and PEA + IOL group were 43.4 ± 12.7 months and 36.5 ± 2.5 months, respectively. LI significantly increased pACD as indicated by the SPAC grade change from 3.8 ± 1.1 to 4.6 ± 1.2 (*p* < 0.001). However, SPAC grade was gradually reduced and reached the pre-LI level by the third year of follow up. PEA + IOL also significantly increased SPAC grade from 6.7 ± 1.6 to 8.7 ± 2.0 (*p* < 0.001), but no time-related change was observed. Twenty-three cases of LI group presented with deterioration of glaucoma control. The type of angle closure, plateau iris configuration, peripheral anterior synechia, and glaucomatous visual field defects were significantly associated with prognosis of glaucoma after LI.

**Conclusions:**

Peripheral ACD is temporarily deepened by LI, but returns to the pre-LI level in approximately three years. The type of angle closure and some factors may be related to glaucoma prognosis after LI.

## 1. Introduction

Angle closure glaucoma (ACG) is prevalent in Asia, and its prognosis is reported to be poorer than that of open angle glaucoma [1–4]. It is well known that anterior chamber configuration, including anterior chamber depth (ACD) and angle openness, is strongly related to the onset of ACG [5–7].

Peripheral laser iridotomy (LI) and lens extraction are currently recommended for the treatment of eyes with angle closure. LI is relatively easy, safe, and an effective procedure for the purposes of prophylaxis and the prevention of ACG progression. Many studies have indicated that LI deepens ACD, particularly in the peripheral area [8–12]. In contrast, some ophthalmologists recommend lens extraction because this procedure could provide a cure [13–15]. Some papers have reported poor long-term prognosis [16, 17] and the lack of effect of LI on the onset of ACG [18]. Many of previous papers focused on the central ACD not peripheral ACD (pACD), although pACD should be more important from regulating aqueous humor outflow than central ACD. In addition, it is not known sufficiently which parameters affect the prognosis of ACG.

We developed a scanning peripheral anterior chamber depth analyzer (SPAC) that measures ACD consecutively from the center to the periphery [19, 20]. In this study, we investigated longitudinal changes in pACD of LI-treated eyes and factors associated with the prognosis.

## 2. Methods

This study was conducted in accordance with the Helsinki Declaration after receipt of approval from the Ethics Committee of Yamanashi University.

## 3. Patients

Patients with primary angle closure (PAC), acute PAC, or chronic angle closure glaucoma (CACG) who were followed up at the University of Yamanashi Hospital were enrolled (LI group). SPAC measurement was performed periodically as well as routine ophthalmic examination, including intraocular pressure (IOP) measurement, slit-lamp examination, fundus examination, best-corrected visual acuity (BCVA) measurement, visual field test, and gonioscopy.

Angle openness was evaluated on the basis of a previous report [21]. In brief, an angle with no visibility of the trabecular meshwork three or more than three-quarters of the angle was diagnosed as an occludable angle. Eyes with an occludable angle but without IOP elevation of more than 21 mmHg or peripheral anterior synechia (PAS) were classified as primary angle closure suspect (PACS). Eyes with an occludable angle and an IOP elevation of more than 21 mmHg or PAS formation were classified as PAC. Eyes with an occludable angle and glaucomatous optic nerve atrophy showing corresponding visual field defect were classified as primary AGC (PACG). Eyes with an occludable angle and an acute IOP elevation of more than 40 mmHg, acute visual acuity loss, ocular pain, and conjunctival injection were classified as acute PAC. Eyes with signs of PACG but no history of acute PAC were classified as CACG.

Patients were excluded from the study if they satisfied any of the following criteria: any signs of secondary angle closure, a history of incisional surgery, or treatment with pilocarpine eye drops after LI.

Patients who had phacoemulsification and intraocular lens (PEA + IOL) insertion without any intra- or postoperative complications were enrolled as controls (PEA + IOL group). Their pACDs were evaluated pre- and post-PEA + IOL surgery by SPAC over a three-year period.

A subject was excluded from the study if he/she satisfied any of the following criteria: having any ocular diseases influencing pACD, treatment with pilocarpine eye drops, visual acuity of less than 20/30, corneal disorders that interfere with the measurement, poor fixation to fixation light, or poor cooperation during SPAC measurements. If both eyes satisfied the entry criteria, the right eye was chosen for the study.

### 3.1. Ophthalmic Examination

Refractive error and BCVA were measured by the medical staff, and slit-lamp examination, IOP measurement, and fundus examination were performed by glaucoma experts. IOP was measured with a Goldmann applanation tonometer. Gonioscopy was performed in a dark room with a Goldmann 2-mirror lens (Haag-Streit, Koeniz, Switzerland). Care was taken to avoid illuminating light on the pupil during gonioscopy. The angle in each quadrant was graded using the Shaffer grading system [22]. The extent of PAS of acute PAC was evaluated as soon as possible after reducing the acute IOP elevation, when corneal edema hindered evaluation of the angle before LI. The visual field test was conducted with a Humphrey field analyzer (HFA) (Zeiss, Dublin, CA).

### 3.2. SPAC Measurement

Experienced operators conducted the measurements in a dark room (approximately 2–3 lux at the level of the patient's eye). The patient was requested to gaze at a fixation light during the measurements. A self-judging program was installed in the SPAC. If an unreliable measurement was made by the SPAC, the measurement was automatically repeated until reliable results were obtained. The principles of SPAC measurement and the data analyses are described elsewhere [19, 20]. In brief, SPAC examines the region from the optical axis to the temporal limbus in approximately 0.66 second, taking 21 consecutive slit-lamp images at 0.4 mm intervals. The camera-captured cross-sectional slit-lamp images are immediately subjected to the analyses, and the radius of curvature, the corneal thickness, and the ACD values are displayed. The SPAC yields numeric and categorical grades that are calculated by comparison with the ACD values derived from a sample of Japanese subjects [19, 20]. The range of ACD values of the patients was divided into 12 groups, each representing an equal increment in the ACD. Group 12 consisted of eyes with the deepest mean ACD values, whereas eyes with the shallowest mean ACD values were allocated to group 1. SPAC measurement was performed within one month before LI or PEA + IOL, within six months after LI or PEA + IOL, and every year thereafter.

### 3.3. Investigated Parameters

The objective variable was the change in SPAC grade and the explanatory variables were age, sex, type of angle closure, SPAC grade, IOP, BCVA, the extent of PAS, the number of antiglaucoma eye drops, and mean deviation (MD) of HFA.

### 3.4. Definition of Poor Prognosis of Glaucoma

Eyes with PAC or CACG that satisfied one or more of the following conditions were judged to show poor prognosis of glaucoma during the follow-up period: two consecutive IOP elevations of more than 21 mmHg or two consecutive IOP elevations of more than 30% of IOP before LI, an increase in the number of antiglaucoma eye drops used for more than one month, additional prescription of oral antiglaucoma medication for more than one month, significant deterioration of MD slope or/and PSD slope for no reason except the progression of glaucomatous optic nerve atrophy, and glaucoma surgery.

Since eyes with acute PAC showed significant variation of IOP and medication in the perioperative period of LI, conditions were judged to show glaucoma progression during the follow-up period were significant deterioration of MD slope or/and PSD slope for no reason except the progression of glaucomatous optic nerve atrophy or glaucoma surgery during the follow-up period.

### 3.5. Statistical Analysis

Data were analyzed with JMP 8.0 software (SAS Institute Inc., Cary, NC), and values are presented as means ± standard deviation. Mann–Whitney's *U* test, one-way ANOVA, and chi-squared test were employed. The association between risk factors and prognosis for glaucoma was evaluated using logistic regression models to determine the odds ratio (OR) and the 95% confidence interval (CI).

## 4. Results

### 4.1. Patients

Forty-eight eyes of 48 subjects with angle closure were enrolled in the LI group (Table 1). There were thirteen male patients and thirty-five female patients, and their mean age was 69.8 + 8.5 years. Mean follow-up time was 43.4 + 12.7 months (range: 24–70 months). Twenty-one eyes of 21 subjects with PEA + IOL were enrolled as controls (PEA + IOL group). There were eleven male patients and ten female patients, and their mean age was 65.6 + 12.7 years. Comparing the preoperative characteristics of the two groups, significant differences were found in the gender distribution, the initial SPAC grade, and the extent of PAS. Thirteen eyes (27.1%) of the angle closure group had plateau iris configuration. The numbers of eyes having plateau iris configuration in PAC, acute PAC, and CACG patients were 4 (14.3%), 2 (40.0%), and 7 (46.7%), respectively. No eyes with plateau iris configuration showed IOP elevation by pupil dilation as a provocative test with topical tropicamide administration after LI.

### 4.2. Early Changes in SPAC Grade by LI

**Figure 1:**
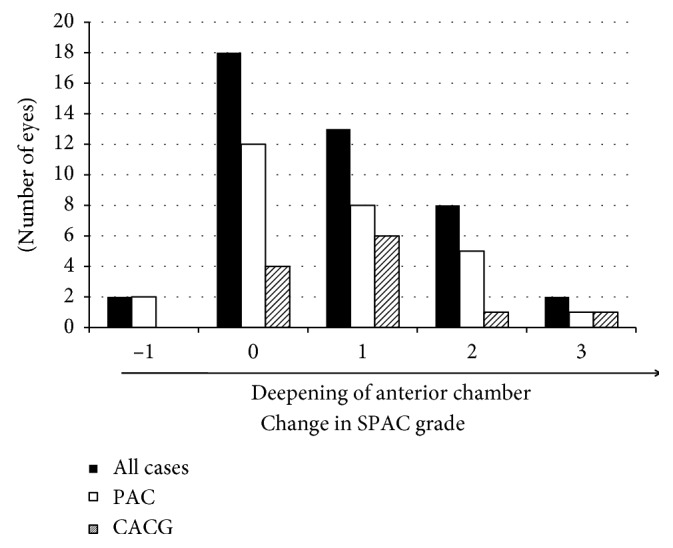
Early changes in SPAC grade by LI. PAC: primary angle closure, CACG: chronic angle closure glaucoma, SPAC: scanning peripheral anterior chamber depth analyzer.

Of the 48 angle closure eyes, five were eliminated from this analysis because of lack of paired SPAC data at pre-LI and six months after LI. SPAC grade before LI was 3.8 + 1.1. LI significantly increased SPAC grade to 4.6 + 1.2 (*p* < 0.001). Mean change was 0.77 + 0.97 (95% CI: 0.47 to 1.07).  Figure 1 shows the distribution of SPAC grade change by LI. Twenty-three eyes (53.5%) exhibited an SPAC grade increase of one or more. Eighteen eyes (41.9%) did not show any SPAC grade changes, and only two eyes (4.6%) showed a decrease in SPAC grade by one. As the number of acute PAC eyes was only two for this analysis, we compared the difference in the distribution of SPAC grade change between PAC eyes and CACG eyes, but found no significant difference. In contrast, LI did not significantly change central ACD. Central ACDs before and after LI were 2.31 ± 0.39 mm and 2.41 ± 0.38 mm, respectively (*p*=0.28).

### 4.3. Long-Term Change in ACD by LI

**Figure 2:**
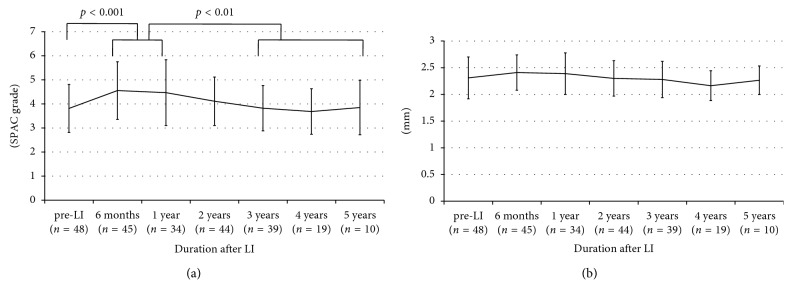
Long-term changes in SPAC grade and central ACD after LI. (a) Long-term changes in SPAC grade, (b) long-term changes in central ACD. SPAC: scanning peripheral anterior chamber depth analyzer, ACD: anterior chamber depth, LI: laser iridotomy. One-way repeated measures ANOVA was employed.

SPAC grade was significantly increased at six months after LI, but tended to gradually decrease during the follow-up period. SPAC grade measured three years after LI was almost the same as that measured at pre-LI, and no further reduction of SPAC grade was recognized after the three-year follow-up period (Figure 2(a)). In contrast, central ACD did not show any significant changes during the follow-up period, although the changing trend seemed to be the same as that of SPAC grade (Figure 2(b)).

### 4.4. Long-Term Change in SPAC Grade by PEA + IOL

**Figure 3:**
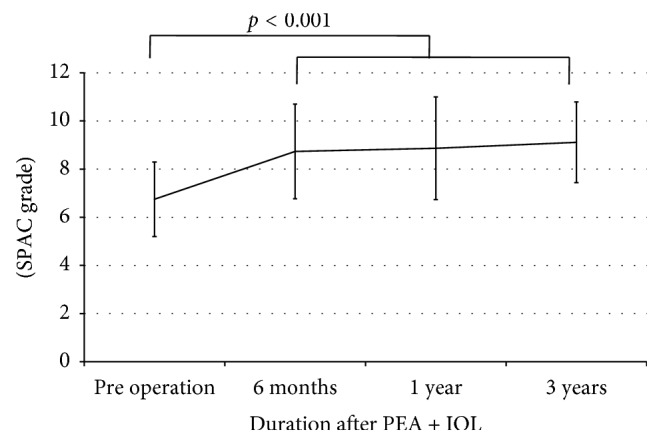
Long-term changes in SPAC grade after PEA + IOL. SPAC: scanning peripheral anterior chamber depth analyzer, PEA + IOL: phacoemulsification and intraocular lens, LI: laser iridotomy. One-way repeated measures ANOVA was employed (*n* = 21).

PEA + IOL significantly increased SPAC grade from 6.8 ± 1.6 to 8.7 ± 2.0 at six months after the surgery (*p* < 0.001). No further changes in SPAC grade were observed during the follow-up period (Figure 3).

### 4.5. Comparison among PAC, Acute PAC, and CACG Eyes

**Figure 4:**
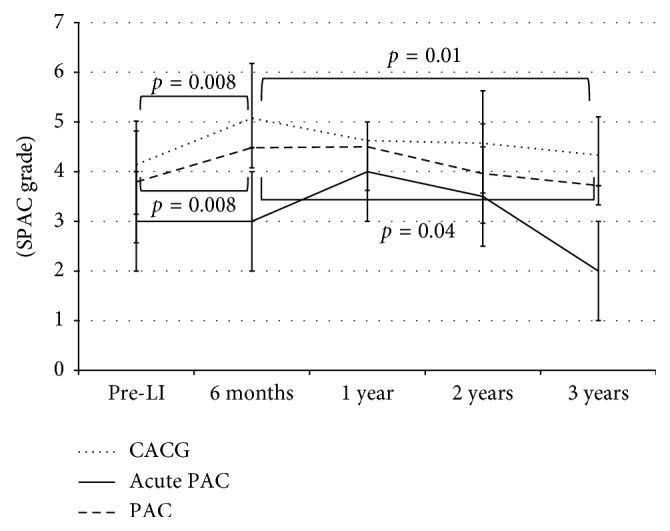
Comparison of SPAC grade and central ACD change profiles among PAC, acute PAC, and CACG eyes. PAC: primary angle closure, CACG: chronic angle closure glaucoma, SPAC: scanning peripheral anterior chamber depth analyzer, LI: laser iridotomy. One-way ANOVA was employed.

Table 2 compares the characteristics of PAC, acute PAC, and CACG eyes before LI. There were no significant differences in age and sex distribution. Acute PAC eyes had the highest IOP, followed by CACG eyes and PAC eyes. Acute PAC eyes had significantly shallower central ACD than the other two types of glaucoma eyes. Acute PAC eyes and CACG eyes demonstrated a significantly greater extent of PAS than PAC eyes.  Figure 4 shows the profiles of SPAC grade change during the follow-up period.

The SPAC grade of PAC eyes and CACG eyes was significantly increased at six months after LI, but returned to the SPAC grade before LI and approximately three years after LI. In contrast, the SPAC grade of acute PAC eyes was not increased by LI six months after LI and was lower than that before LI and three years after LI, although the difference was not significant. All the three types of eyes did not show any significant changes of central ACD during the follow-up period.

### 4.6. Association between Poor Prognosis and Glaucoma Type

**Figure 5:**
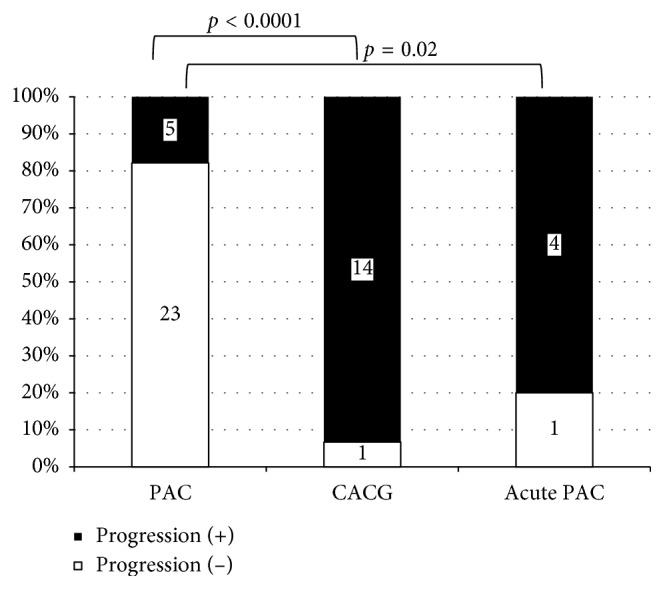
Comparison of prognosis among PAC, acute PAC, and CACG eyes. PAC: primary angle closure, CACG: chronic angle closure glaucoma, SPAC: scanning peripheral anterior chamber depth analyzer. The chi-squared test was employed.

Twenty-three eyes (47.9%) satisfied the criteria for poor prognosis. Only five PAC eyes (17.9%) demonstrated poor prognosis, whereas four acute PAC eyes (80.0%) and 14 CACG eyes (93.3%) showed poor prognosis. There was a significant difference in results among PAC, acute PAC, and CACG eyes (Figure 5).  Table 3 shows the results of comparison of parameters between eyes with good prognosis and those with poor prognosis. Plateau iris configuration, greater extent of PAS, and worse MD value were chosen as the significant parameters. Four (16.0%) eyes had plateau iris configuration among the eyes with good prognosis, whereas nine (39.1%) had plateau iris configuration among the eyes with poor prognosis.

### 4.7. Difference in Anterior Ocular Configuration between PAC Eyes with Good Prognosis and Those with Poor Prognosis

**Figure 6:**
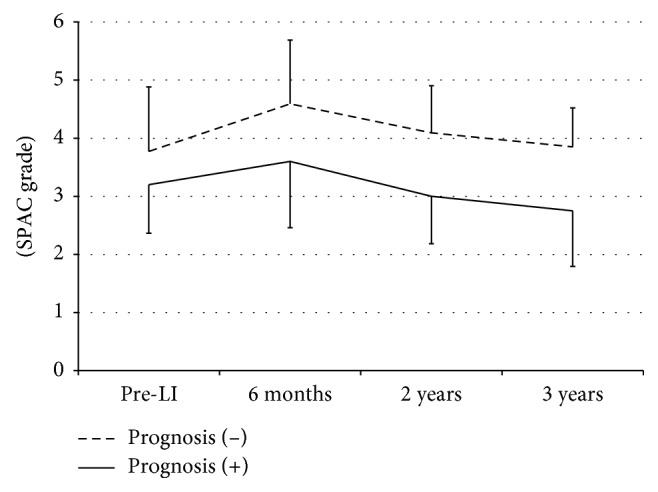
Comparison of SPAC grade change profiles between PAC eyes with good prognosis and those with poor prognosis. SPAC: scanning peripheral anterior chamber depth analyzer, LI: laser iridotomy. One-way ANOVA showed no significant difference. *p* values were calculated by Mann–Whitney's *U* test.

As the majority of CACG eyes and acute PAC eyes showed poor prognosis, only PAC eyes were subject to this investigation. There was tendency of significant differences in all the investigated parameters between PAC eyes with poor prognosis and those with good prognosis, but SPAC grade of eyes with good prognosis was always greater than that of eyes with poor prognosis, and the deepening of ACD was more apparent in eyes with good prognosis (Figure 6).

## 5. Discussion

There are few reports of the longitudinal changes in ACD after LI [16]. In this study, we investigated quantitatively the longitudinal changes in ACD after LI and the factors associated with the prognosis. LI significantly increased pACD but not central ACD, similar to previous reports [8, 10, 12, 17, 23–27]. The increase in pACD by LI was reduced gradually and returned to near its original level at three years after LI.

We investigated also the ACD changes of eyes that underwent PEA + IOL for comparison. PEA + IOL increased ACD, and this effect was sustained for three years. The effect of LI on pACD is temporary, while that of PEA + IOL on pACD may be much more sustained. The current results also demonstrated that SPAC successfully evaluated ACD changes objectively and quantitatively.

LI is widely used to treat eyes with an occludable angle. Previous studies have indicated that LI indeed deepens pACD and widens the occludable angle [10, 12, 16, 17], making it a good treatment choice for ACG. However, it has been reported that the long-term prognosis of some eyes with angle closure is not always good [16, 28–30]. Although previous studies have identified the risk factors associated with glaucoma progression [16, 28], the current study clarified that some risk factors, including plateau iris configuration, extent of PAS, and MD value, could be associated with the deterioration of glaucoma.

Our study revealed that acute PAC eyes and CACG eyes showed poor prognosis compared to PAC eyes. Although there was no significant difference, PAC eyes with lower SPAC grade seemed to have a greater tendency to develop glaucoma. Plateau iris configuration also contributed to the poor prognosis, consistent with a previous report [31]. As the number of patients was relatively small in this study, further investigations employing a larger number of subjects are necessary to clarify these points. Some guidelines recommend LI for acute PAC. LI certainly reduces IOP but our study indicates that the long-term prognosis is poor. The deepening of pACD by LI was less obvious in eyes with acute PAC than in eyes with PAC or CACG. The effect of LI on acute PAC eyes may be temporary. It may be better to consider lens extraction or other treatments as an option even after LI.

The current study also revealed that 93.3% of CACG eyes showed poor prognosis during the long-term follow up, accompanied by a decrease in pACD, which means that LI alone had some limitations when used to treat CACG eyes. In contrast, only 17.9% of PAC eyes exhibited poor prognosis. It should be noted that there were no significant differences in SPAC grade and change profile between CACG eyes and PAC eyes. We consider two reasons for these differences. First, the criteria for LI were not the same between CACG eyes and PAC eyes. We sometimes perform LI for CACG eyes, but there are no established criteria recommending LI for the treatment of PAC eyes. If the contralateral eye had a history of acute PAC, LI should be performed. However, the ophthalmologist in charge could decide whether or not LI should be performed on the basis of the patient's willingness to undergo LI. Second, the current results clearly showed a significant difference in pACD between acute PAC eyes and CACG eyes, which may indicate differences in the mechanisms and the related factors of onset of these two types. Eyes with PAC in this study may develop either acute PAC or CACG without acute PAC. Therefore, pACD of CACG eyes was wider than that of PAC eyes. We currently indicate that the effect of LI on anterior chamber configuration from a point of glaucoma subtype view. Han et al. approached this subject from a biostructural point of view. They reported lower ACD, higher lens vault, and lower angle open distance were deeply associated LI- induced changes in anterior segment configuration [32], which is consistent with the current study.

Many papers have described the risk factors for the development of ACG, including uveal effusion, iris thickness, anterior rotation of ciliary body, and lens vault [7, 33, 34].

However, many of these still remain unexplored. The current results clearly indicate that it is absolutely recommended to screen eyes at risk of ACG at an early a stage as possible. However, there are no clear criteria for the recommendation of LI or lens extraction for PAC eyes. The current results may indicate that it was not necessary to perform LI in some eyes with PAC. Although Azuara-Blanco et al. reported that clear-lens extraction showed greater efficacy and was more cost-effective than laser peripheral iridotomy [15], we sometimes experience difficult cases to perform lens extraction and severe complications, and some patients hesitate to receive clear lensectomy as a treatment for their angle closure glaucoma. It is necessary to establish guidelines for the treatment of eyes with angle closure.

We employed subjects with PEA + IOL as controls in this study. The ACD of these subjects was greater even before the surgery. We have to recognize that long-term changes in ACD of these subjects are not always the same with those with LI surgery.

In summary, LI increased pACD, but the increased pACD returned to the baseline in approximately three years. Although the reduction of pACD may be related to glaucoma progression after LI, other risk factors should be considered. LI did not improve prognosis in some cases, particularly in eyes with acute PAC and CACG. Quantitative observation of the longitudinal changes in pACD may be necessary for the management of ACG.

## Figures and Tables

**Table 1:** Patient's profile.

	LI group	PEA + IOL group
Total number of patients	48	21
Age (years)	69.8 ± 8.5	65.6 ± 12.7
No. of female (%)	35 (72.9)	10 (47.6)^*∗*^
PAC	28	0
Acute PAC	5	0
CACG	15	0
Extent of PAS (%)	13.5 ± 21.8	0^*∗*^
Initial SPAC grade	3.8 ± 1.1	6.8 ± 1.6^§^

LI: laser iridotomy, PEA + IOL: phacoemulsification and intraocular lens, PAC: primary angle closure, CACG: chronic angle closure glaucoma, PAS: peripheral anterior synechia, SPAC: scanning peripheral anterior chamber depth analyzer. ^*∗*^*p* < 0.001 (chi-squared test), ^§^*p* < 0.0001 (Mann–Whitney's *U* test).

**Table 2:** Comparison among types of angle closure.

	PAC	Acute PAC	CACG
Number of eyes (no. of plateau iris configuration)	28 (4)	5 (2)	15 (7)
Age (years)	69.5 ± 9.1	68.6 ± 11.5	70.4 ± 6.22
No. of female (%)	23 (79.3)	4 (80.0)	8 (57.1)
IOP before LI (mmHg)^*∗*^	15.4 ± 2.7	64.6 ± 11.7	19.4 ± 4.3
Initial SPAC grade^*∗∗*^	3.8 ± 1.1	3.0 ± 1.2	4.1 ± 0.7
Initial central ACD (mm)^§^	2.28 ± 0.36	1.90 ± 0.29	2.53 ± 0.36
Extent of PAS (%)^§^	4.4 ± 6.5	22.5 ± 28.7	30.0 ± 30.4

LI: laser iridotomy, PAC: primary angle closure, CACG: chronic angle closure glaucoma, IOP: intraocular pressure, SPAC: scanning peripheral anterior chamber depth analyzer, ACD: anterior chamber depth, PAS: peripheral anterior synechia. ^*∗*^*p* < 0.001 (one-way ANOVA), ^*∗∗*^*p* < 0.01(chi square test), ^§^*p* < 0.0001 (one-way ANOVA).

**Table 3:** Risk factors associated with prognosis.

Prognosis	Age (yrs)	% Of plateau iris configuration	Extent of PAS (%)	Pre-ACD (mm)	Pre-grade	MD value
Good (*n* = 25)	69.0 ± 9.4	8.0	4.6 ± 1.1	2.3 ± 0.4	3.7 ± 1.3	−0.8 ± 2.04
Poor (*n* = 23)	70.4 ± 14.5	39.1	23.3 ± 27.5	2.4 ± 0.6	3.9 ± 0.9	−4.3 ± 4.4
Relative risk (95%CI)		2 (1.26–3.17)	1.07 (1.02–1.16)/1°^§^			1.62 (1.16–2.64)/−1 dB^§^
*p* value	0.57	0.02	0.008	0.41	0.53	0.005

PAS: peripheral anterior synechia, ACD: anterior chamber depth, MD: mean deviation, CI: confidence interval. §: unit relative risk. Logistic regression analysis was employed.

## Data Availability

The data used to support the findings of this study are available from the corresponding author upon request.
